# Rescue Stenting for Inadvertent Branch Vessel Occlusion Following Cerebral Aneurysm Embolization With the Woven EndoBridge Device

**DOI:** 10.7759/cureus.59880

**Published:** 2024-05-08

**Authors:** Reyna Escalante, Colin Son

**Affiliations:** 1 Neurosurgery, University of the Incarnate Word School of Osteopathic Medicine, San Antonio, USA; 2 Neurosurgery, Neurosurgical Associates of San Antonio, San Antonio, USA

**Keywords:** neurointerventional surgery, woven endobridge device, stroke, neuroendovascular, brain aneurysm

## Abstract

Intrasaccular flow modification with devices like the Woven EndoBridge (WEB, MicroVention, Inc., Aliso Viejo, California, US) is an increasingly utilized endovascular treatment for bifurcation aneurysms. Among the potential complications of the procedure is branch vessel occlusion. There are no previous publications of rescue stenting for inadvertent branch vessel occlusion with the WEB device. We report two cases of rescue stenting following branch vessel occlusion after cerebral aneurysm embolization with the WEB device. In both cases, rescue stenting with a Neuroform Atlas stent Stryker, Fremont, CA, US) successfully revascularized the occluded vessel and led to good patient outcomes.

## Introduction

Intrasaccular flow modification with devices like the Woven EndoBridge (WEB, MicroVention, Inc., Aliso Viejo, CA, USA) is an increasingly utilized treatment for endovascular treatment of wide-necked bifurcation aneurysms. The WEB device, in particular, has a remarkably low complication rate across publications [1-3]. This may be more pronounced as compared to alternative endovascular treatment options for these aneurysms [4-6].

But as with all aneurysm treatment options, some risks exist. As a truly intrasaccular device by design, the WEB potentially holds a lower risk of ischemic complications as compared to stent-assisted embolization or flow diversion [7]. But as compared to more conventional intra-aneurysmal treatments, namely, coiling, the placement of the WEB in three-dimensional space within the aneurysm can sometimes be difficult to ascertain on two-dimensional biplane fluoroscopy [8]. This can be a contributing factor to the misplacement of the WEB. The WEB device indications for use (IFU) recommend tolerating just short of 50% coverage of branch vessels by device protrusion outside the aneurysm [9]. Coverage of branch vessel origin beyond that carries a risk of flow limitation or thrombotic complications.

In such scenarios when the WEB has been detached and parent vessel occlusion occurs, the rescue treatment options are not clear. Here, we present two of the first such cases of misplaced WEB devices with resultant iatrogenic parent vessel occlusions with rescue stenting with Neuroform Atlas stents (Stryker, Fremont, CA, US).

## Case presentation

Case one

**Figure 1:**
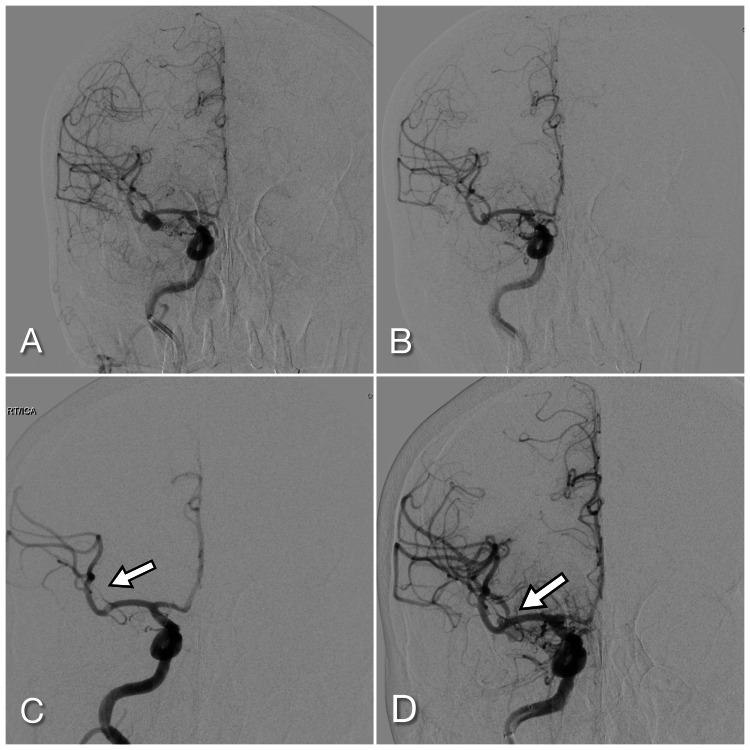
Right middle cerebral artery aneurysm pre- (A) and immediately post-embolization with the WEB device; (B). On return to the angiosuite with M2 branch occlusion (C) and reopened post stenting (d) WEB: Woven EndoBridge (MicroVention, Inc., Aliso Viejo, California, US)

The first patient was a 71-year-old female with an incidental 8.4 mm right middle cerebral artery aneurysm (Figure 1A) who, following a discussion on observation versus open surgical treatment versus endovascular treatment elected for the latter. The treatment was carried out under general anesthesia via a transradial approach with a 6 Fr Benchmark catheter (Penumbra, Alameda, CA, USA) placed in the right internal carotid artery. The patient was on daily aspirin 81 mg pre-operatively. She received 50 units/kilogram of intravenous heparin on arterial access.

The decision was made to plug the neck origin of the aneurysm with a WEB SL 4 mm x 3 mm device and this was done with a Via 17 microcatheter (MicroVention) in standard fashion. There was nearly complete aneurysm occlusion immediately (Figure 1B). Prior to detachment, multiple oblique views of the WEB device and aneurysm were taken with no concern for branch vessel compromise and completely normal flow through all parent vessels. No three-dimensional imaging was obtained with the device in place. The device was detached and the access catheters were removed. The patient was extubated and found at her neurological baseline. The heparin was not reversed.

Approximately 45 minutes post-procedure while in the post-anesthesia recovery area, the patient developed left-sided weakness through the arm and face. She was taken immediately back to the angiosuite with no intervening non-invasive imaging.

Repeat angiogram showed the WEB device in a stable position and the aneurysm occluded but with a new posterior M2 branch occlusion (Figure 1C). Additional oblique projections appeared to show the origin of this M2 branch partially obstructed by the WEB device itself. The patient was dosed with a half cardiac bolus dose of tirofiban. Using an SL-10 microcatheter (Stryker), the occluded branch was accessed. The occlusion was reopened with a Neuroform Atlas 3 mm x 21 mm stent. She was extubated post-procedure and started on a low-dose heparin drip and loaded with 300 mg of clopidogrel via a placed nasogastric tube approximately two hours after the tirofiban dose.

Immediately post-procedure, her left-sided weakness was markedly improved and she was back to her neurological baseline by the next morning. She was discharged on post-operative day 2 on a dual antiplatelet regimen with twice a day clopidogrel 75 mg added to her already existing aspirin. At 13 months, she was modified Rankin scale of 0 and the aneurysm remained occluded by repeat angiogram at three months and then magnetic resonance angiography (MRA) at the one-year mark.

Case two

**Figure 2:**
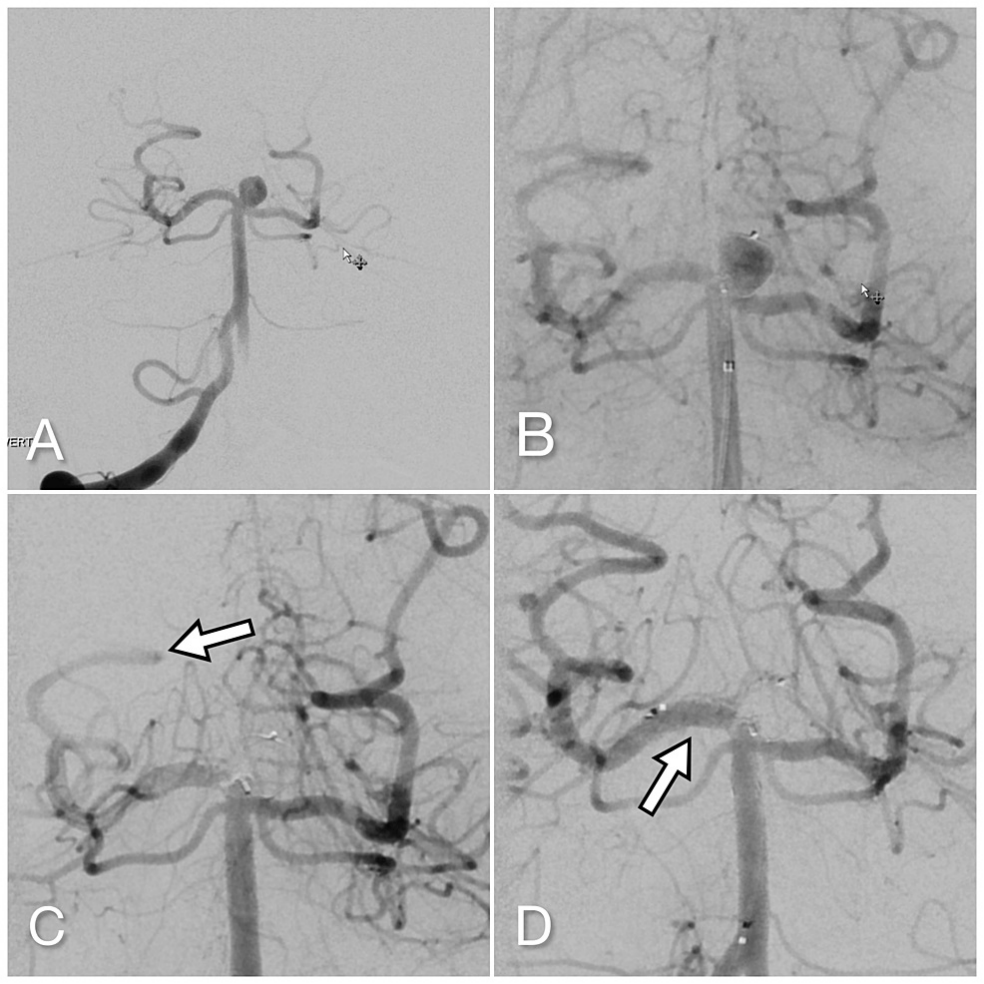
Basilar tip aneurysm pre- (A) and immediately post WEB deployment (B); Delayed angiogram showing decreased flow in the right posterior cerebral artery (C) but restored following rescue stenting (D) WEB: Woven EndoBridge (MicroVention, Inc., Aliso Viejo, California, US)

The second patient was a 65-year-old male with an asymptomatic, unruptured 8 mm basilar tip aneurysm (Figure 2A) who, after discussion, elected for endovascular treatment. Again, the patient was on preoperative aspirin 81 mg per day. The procedure was performed under general anesthesia from a transradial approach with a 6 Fr Benchmark catheter advanced into the distal right vertebral artery. On radial access, he was dosed with 50 units/kilogram of intravenous heparin.

Based on multiple projections, a WEB SL 8 mm x 5 mm was decided on for embolization. The device was delivered through a Via 27 microcatheter (MicroVention). Again the WEB device placement was investigated with multiple angiographic runs in multiple oblique projections prior to detachment and felt to be in a good position. There was an initial substantial decreased flow into the aneurysm (Figure 2B).

Post device detachment, final angiography was performed routinely with no expectation of any changes. However, it was noted that there was slower antegrade flow now into the right posterior cerebral artery (Figure 2C). As the aneurysm itself continued to occlude and less contrast was within the WEB device itself, it became apparent that the device was covering the origin of the right posterior cerebral artery.

As in the first case, the patient was now dosed with a cardiac half-loading dose of tirofiban. A Via 17 navigated easily past the device and into the right P1. A Neuroform Atlas 3 mm x 15 mm stent was then deployed from here back into the basilar artery (Figure 2D). The flow had been reestablished on repeat angiogram with no obvious distal thromboemboli. The heparin was not reversed. The patient was extubated and found without any new neurological deficit. He was maintained on a low-dose heparin drip and approximately three hours later, a tirofiban dosing of 150 mg of clopidogrel was given. He was started on clopidogrel 75 mg twice a day and discharged on post-operative day 1.

On repeat angiogram at nearly four months, the aneurysm was completely occluded and the stent and right posterior cerebral artery open. At 11 months, he remained a modified Rankin scale of 0.

## Discussion

Embolization with the WEB device is very low risk, especially in on-indication aneurysms as compared to alternative endovascular options. Still, there are many interesting case reports of complications of misplaced or migrated devices. Several case reports exist of WEB devices that changed position post detachment resulting in parent vessel compromise and treated with rescue stenting [10-12].

More extreme device complications reported include post-detachment complete WEB extrusion from the aneurysm into branch vessels. Reported rescue techniques here include snare and alligator clamp retrieval in addition to stenting to tack the migrated device up against the vessel wall and maintain antegrade flow [11,13,14].

In addition to these previous reports of rescue stenting in the case of misplaced WEB devices, there exist reports of intent to treat with balloon-assisted WEB placement [15] and of intent to treat with stent-assisted WEB embolization with success with a Neuroform Atlas stent [16].

Our two cases of rescue stenting add to the knowledge base regarding the interaction of the WEB device and various neurovascular stents. The Neuroform Atlas stent has enough radial force to reposition the WEB device and it, along with several previously reported stents, is a viable option for the revascularization of parent vessel occlusions caused by the WEB device.

Previous reports of parent vessel compromise have focused on a post-device detachment change in position. Our cases are less excusable, with simple poor recognition of the device position before detachment. A 3D rotational angiogram may assist in fully understanding the device position in complex aneurysm/parent vessel anatomy prior to detachment [8]. It has become standard practice in our practice following these cases.

This case series is limited by its small sample size and single-operator experience.

## Conclusions

Parent vessel occlusion is a potentially devastating complication of embolization with the WEB device. In such cases, rescue stenting of branch vessel occlusion is feasible with the Neuroform Atlas stent, which has the radial force to reposition the WEB device and recanalize target vessels.
